# Medicines prices in International (Geary–Khamis) Dollar. The comparison between regulated and deregulated markets

**DOI:** 10.1371/journal.pone.0304400

**Published:** 2024-06-07

**Authors:** Tomasz Zaprutko, Julia Cynar, Maria Sygit, Aleksandra Stolecka, Patrycja Skorupska, Paulina Jaszcz, Dorota Kopciuch, Anna Paczkowska, Piotr Ratajczak, Krzysztof Kus

**Affiliations:** 1 Department of Pharmacoeconomics and Social Pharmacy, Poznan University of Medical Sciences, Poznań, Poland; 2 Student Scientific Society, Department of Pharmacoeconomics and Social Pharmacy, Poznan University of Medical Sciences, Poznań, Poland; Hosei University: Hosei Daigaku, JAPAN

## Abstract

**Background:**

Affordability of medicines is key for effective healthcare. Thus, we compared medicine prices using International Dollar (I$), which allows confronting the values of different currencies. Besides, we intended to verify if pharmaceutical market deregulation leads to lower medicines prices.

**Materials and methods:**

We conducted the study between December 2019 and September 2022 collecting data from 21 countries. From the preliminary sampling of 30 medicines, we selected 10 brand names (5 Rx and 5 OTC brands) for the analysis. In each country, we collected price information from 3 pharmacies and then converted them to the I$ using the rates published by the International Monetary Fund.

**Results:**

There were differences between regulated and deregulated markets in prices presented in I$. For instance, Aspirin C® (10 soluble pills) was on average I$ 5.41 in Finland (regulated market) and I$ 13.25 in Brazil. The most expensive Xarelto® 20 x 28 pills (I$ 538.40) was in Romania, which in the case of other medicines, was in the group of cheaper countries. There was no statistical significance in price comparison between regulated and deregulated markets. In some cases, however, regulated markets offered lower prices of the same medicine than deregulated markets.

**Conclusion:**

The analysis revealed differences in I$ prices between countries. Pharmaceutical market regulation does not mean higher prices of medicines. There is a need for affordable medicines. Hence, decision-makers should work on the medicines prices and adjust them to the local economies. I$ could be important in creating pharmaceuticals prices, and the conducted study should encourage other researchers to present their results using this currency.

## 1. Introduction

Access to medicines and their affordability are key factors for effective healthcare [1–3]. However, even one-third of the global population can not reach medications. It results, for instance, from price barriers, medicine shortages, and internal market regulations [4–7]. The non-accessible non-affordable medicines, and delayed market launch of medicines concern not only low and middle-income countries but also the wealthiest parts of Europe, the USA, or Australia [8, 9].

Medicines prices vary globally [8]. Sometimes, these differences seem surprising and e.g., Brekke et al. revealed lower prices in Norway compared to other European countries [10]. Price differences might result from several facts. Pharmaceutical pricing is adjusted nationally and different price levels are the result of various national policies [11]. Moreover, differences in medicines prices may also result from external price referencing (EPR) [8], price cuts [11], or internal market regulation [7, 12].

Differences in medicine prices contribute to the development of the concepts like free riding, which explains that medicine prices are higher in the US than in other markets. This stems from the belief that richer countries spend more per capita on pharmaceuticals paying for R&D [2, 13]. However, studies also present higher medicines prices in poorer countries than in richer ones [2]. On the one hand, it allows to discuss several medicine pricing phenomenons. On the other, various methods for comparing drug prices frequently contribute to remarkably different results [4]. Nonetheless, as revealed by Mardetko et al. [14] and Daalen et al. [15] there is still a paucity of analyses on actual medicine prices with limited insight into drug pricing mechanisms, which makes it difficult to compare drug prices. Hence, it is important to study prices in a possibly comparable way, which should be focused on real access and affordability of medicines.

Considering these facets, we compared medicines prices using the International Dollar (I$). I$ allows the comparison of the values of different currencies, and analyses between countries have been adjusted to reflect currency exchange rates, purchasing power parity (PPP), and average goods prices among countries [16, 17]. Moreover, we intended to compare prices between regulated and deregulated markets to verify if pharmaceutical market liberalization leads to lower medicines prices.

## 2. Materials and methods

The study presents the results of the medicines prices analysis conducted between December 2019 and September 2022 in selected countries. Initially, we planned to conduct the analysis within a shorter period, but the COVID-19 pandemic and the lockdowns contributed to the time extension.

We know that several facets or restrictions influence markets, contributing to the difficulties in evaluating if some market is regulated or deregulated [18, 19]. These are, for instance, demographic and geographical restrictions or pharmacy ownership solely by pharmacists [20]. Although we focused more on the above factors, pharmaceutical markets might also be considered in relation to the pricing system. Moreover, e.g., in the European Union (EU), there is no uniform or even predominant model of the pharmacy market [18]. However, some general assumptions distinguish pharmaceutical markets, allowing them to indicate whether they are regulated or deregulated [19]. S1 Table presents basic assumptions of the pharmaceutical pricing system in selected countries to show the complexity of market evaluations and comparisons. Despite potential difficulties, we analyzed data from 21 countries, which we tried to divide following market regulation (15 countries: Poland, Hungary, Belgium, Finland, Germany, Croatia, Austria, Italy, Spain, Portugal, Greece, Romania, Turkey, Indonesia, and Australia) and deregulation (6 countries: Czech Republic, Norway, Switzerland, Japan, Brazil, and the US) [18, 21–25]. From the preliminary sampling of 30 medicines, we selected 10 brand names (5 Rx and 5 OTC brands) for the analysis. Based on our knowledge and experience, we selected frequently used medicines and with potentially high market share. We drew out following Rx brands: Mysimba ®, Cardura XL 4mg ® - 30 tab., Yasmin ®, Preductal MR 35 mg ® - 60 tab., Xarelto 20 mg ® -28 tab.. In the case of OTC brands, these were: Aspirin C ® - 10 soluble tab., Voltaren gel ® - 50 mg, Nurofen express 400 mg ®– 20 tab., Tantum verde spray 1,5mg/ml ® - 30 ml, Mucoslovan 6mg/ml 100 ml ® (The questionnaire used during data collection is presented within supplementary materials–S1 Questionnaire).

We collected information about all analyzed medicines only in 5 (Austria, Italy, Portugal, Poland, and Romania) out of 21 analyzed countries. This means we have gained prices of x or x+1 medicines in some countries, for instance. It could result from the fact that brand names of the same medicine may differ between countries, or alternatively, products with the same brand name may contain different active substances or strengths [26, 27]. Considering these facets, we decided not to exclude countries with partially collected data (the base sheet is presented within supplementary materials–S1 Data). We are aware, however, that it is a study limitation.

We only took into account total pharmacy prices of medicines. It results from the possible impact of various price regulations, which could have a crucial influence on the study results, especially in the case of Rx brands. Despite this, we are aware that we could not avoid the impact of several laws and pricing differences on the results of our study.

Depending on the local market and pharmaceutical law regulations, prices may vary between pharmacies. Hence, in each country, we collected price information from 3 pharmacies. There were no particular inclusion criteria. Thus, it was opportunistic. However, we assumed we would get information from pharmacies located in different districts. We obtained data by direct contact during our stays in analyzed countries or by our relatives and friends living in/visiting included countries.

We adopted the principle of trust in the prices’ accuracy because participants had no purpose in presenting false information. This is due to several factors. Participation in the study was voluntary, and there were no incentives for providing information. We advised potential participants that price information was collected solely for scientificpurposes and that the study was anonymous. Thus, neither their names nor the pharmacy’s name was recorded or disclosed to avoid, among others, accusations of pharmacy advertising, which is strictly forbidden, e.g., in Poland [2].

We collected information about the prices in local currencies, and after that, we converted them to the I$ using the rates published by the International Monetary Fund [28]. Then, we compared the results between countries according to market regulations. We classified analyzed countries into regulated and deregulated markets using market characteristics found in the literature and our own assessment based on the given market rules.

The data were expressed as means, SEM (standard error of the mean), median, minimum, and maximum as numbers. The data distribution pattern was not normal (unlike the Gaussian function). The analysis of the Friedman test determined significant differences between group results for p < 0.05 (using the Bonferroni correction).

What is important, the study was subjected to the opinion of the Ethics Committee, which stated (decision KB-747/23) that this research is not a medical experiment, and according to the Polish law and GCP regulations, does not require approval of the Bioethics Committee. The document was attached when submitting the work to the editorial office. Besides, „inclusivity in global research questionnaire” is presented within supplementary materials–S2 Questionnaire.

## 3. Results

Using I$ allowed us to compare medicine prices unusually. We decided to present the results mainly within the tables (Tables 1 and 2) to make study, in our opinion, reader-friendly. Despite this, we present some of the results within the text likewise.

**Table 1:** The medicines cost (I$) in the selected countries.

Analyzed Medicine	Cost in I$					
	($\bar{x}$) ± SEM		Median^#^	Min^#^	Max^#^	p
	regulated prices (r)	deregulated prices (der)	r/der	r/der	r/der	
**Prescription medicines (Rx)**						
Xarelto 20	168.76 ± 40.32	237.02 ± 0.00	125.52	85.01	538.40	n.d.
(28 tab.)	n = 11	n = 1	237.02	237.02	237.02	(NS)
Yasmin	24.53 ± 3.73	33.94 ± 13.13	19.03	12.51	57.46	0.3543
(21 tab.)	n = 13	n = 5	24.49	3.19	75.14	(NS)
Mysimba	202.87 ± 21.00	115.73 ± 102.43	199.92	111.47	392.72	0.1908
8 mg+90 mg	n = 13	n = 2	115.73	13.30	218.17	(NS)
(112 tab.)						
Cardura XL 4mg	33.19 ± 15.48	55.58 ± 22.78	14.89	3.87	184.63	0.4313
(30 tab.)	n = 11	n = 5	26.05	7.91	126.07	(NS)
Preductal MR 35 mg (60 tab.)	22.60 ± 6.60	5.20± 0.61	18.63	6.22	66.45	0.2433
	n = 8	n = 2	5.20	4.59	5.81	(NS)
Total Rx	95.69 ± 14.39	61.77± 19.74	42.91	3.87	237.02	0.2571
	n = 56	n = 15	25.45	3.19	538.40	(NS)
**Over-the-counter medicines (OTC)**						
Aspirin C	8.45 ± 1.21	8.03 ± 2.53	8.13	0.49	18.12	0.8785
(10 tab.)	n = 15	n = 4	8.89	1.11	13.25	(NS)
Voltaren 50mg	16.29 ± 2.89	10.22 ± 2.48	13.93	3.66	48.51	0.2532
(gel– 1 tube)	n = 14	n = 5	11.76	2.80	17.36	(NS)
Mucosolvan 6mg/ml	9.64 ± 1.32	9.44 ± 3.70	9.97	2.49	15.80	0.9480
	n = 10	n = 3	8.17	3.76	16.39	(NS)
(100 ml. bot.)						
Nurofen express 400 mg	11.35 ± 1.49	10.75 ± 2.89	10.82	4.76	20.77	0.8550
	n = 11	n = 3	8.39	7.36	16.49	(NS)
(20 caps.)						
Tantum Verde 1.5 mg/ml (spray 30ml)	11.82 ± 1.11	12.10 ± 0.44	13.39	4.38	17.06	0.9060
	n = 12	n = 3	11.72	11.59	12.98	(NS)
Total OTC	11.58 ± 0.87	10.01 ± 1.09	10.67	0.49	48.51	0.3655
	n = 62	n = 18	10.49	1.11	17.36	(NS)
Total r/der	51.50 ± 7.84	33.53 ± 9.93	14.89	0.49	538.40	0.2558
	n = 118	n = 33	12.23	1.11	237.02	(NS)

^#^regulated vs deregulated markets

NS–non-significant

**Table 2:** The total medicines cost (I$) in selected countries.

Analyzed medicines	Cost in I$		Median^#^	Min^#^	Max^#^	p
	($\bar{x}$) ± SEM					
	Prescription medicines (Rx)	Over-the-counter medicines (OTC)	r/der	r/der	r/der	
Total cost regulated markets	95.69 ± 14.39	11.58 ± 0.87	42.91	3.87	538,40	<0.0001*
	n = 56	n = 62	10.66	0,49	48,51	(SS)
Total cost deregulated markets	61.77± 19.74	10.00 ± 1.09	25.45	3,19	237.02	0.0072*
	n = 15	n = 18	10.49	1.11	17.36	(SS)
p	0.2571	0.2558				
	(NS)	(NS)				

^#^regulated vs deregulated markets

SS–statistically significant

There were differences in prices presented in I$ within regulated and deregulated markets and between them. For instance, Aspirin C® was on average I$ 5.41 in Finland and I$ 10.09 in Poland, which are known as countries with regulated markets. On the contrary, in Brazil, the price was I$ 13.25.

The analysis also revealed irregularity in pricing trends within a country. For example, in Romania, the price for Aspirin C® (I$ 11.48) was among highest prices for that medicine in our study, contrary to Yasmin ®, which was the second (I$ 12.51) from the end in Romania. Importantly, these medicines are labeled by the same pharmaceutical company. The cost of Xarelto ® turned out to be higher in Romania (I$ 538.40) than in other countries. Interestingly, the average price of Xarelto ® was I$ 168.76 in regulated markets, which was lower than in the example of a deregulated country (I$ 237.02).

In the case of, e.g., Mysimba ®, the price in Romania was I$ 269.34, and it was higher than in Norway and Belgium (I$ 13.30 and I$ 111.47 accordingly) but lower than in Portugal (I$ 392.72), for instance.

The Voltaren gel ® - 50 mg analysis revealed the lowest prices in Norway (deregulated market) and Austria (regulated market). It was I$ 2.80 and I$ 3.66 respectively. Following gross domestic product (at purchasing power parity) per capita, these countries belong to the richest in the world [29]. The highest price was observed in Indonesia (I$ 48.51,) followed by Portugal (I$ 23.98) and Finland (I$ 21.85). On average, the price for Voltaren gel ® - 50 mg was I$ 16.29 in regulated and I$ 10.22 in deregulated markets.

## 4. Discussion

To our knowledge, it was the first study to comparemedicine prices in I$. Among the most important findings, we revealed that prices were in some cases lower in the reachest countries like e.g., Norway than in other analyzed countries. In addition, we observed price fluctuations within a country resulting from the fact that in the exact country one medicine was among the cheapest, and the other was in the group of most expensive in our study. For example, we found Romania among the cheapest countries in the case of Yasmin ® (I$ 12.51; $\bar{x}$ = 27.15), but Xarelto ® (I$ 538.4; $\bar{x}$ = 174.45) was the most expensive there. We also revealed that market regulation does not mean higher prices than in deregulated markets (no statistical significance in analyzed medicines). In our study, e.g., Voltaren gel ® - 50 mg tourned out to be, on average, more expensive in regulated markets. However, the analysis of individual countries provided interesting information. Although Austria, Australia, and Poland were in the group of regulated markets, the price for Volatren ® was lower than in most deregulated countries.

Although access to medicines is a fundamental human right, the price is frequently the main barrier to getting them [1]. Hence, the results confirm the need for medicine price adjustments in local economies, guaranteeing affordability. The study also corroborates, e.g., the space for negotiations aimed at affordablemedicines for individual countries and justifies such phenomenons like EPR, which is applied in many countries [30]. It is noteworthy that EPR provides some benchmarks for policy-makers and savings. However, it may simultaneously delay the product launch in countries where medicine prices are generally low [9]. Nonetheless, the scope, relevance, and methodological design of EPR may differ across countries [9].

However, prices of medicines may vary forvarious reasons including, for instance, differences on value-added tax on medicines [11, 31]. There are also local and sometimes confidential discounts from pharmaceutical companies and wholesalers [8]. Pharmacies could benefit from several rebates likewise. Market competition also contributes to price cuts or establishing lower pharmacy margins. Hence, there are possible differences in prices of the same medicine within a country, which we also observed in our study. For example, in Romania, the lowest offer for Aspirin C® was I$ 6.29 and the highest was I$ 16.40. In Finland, however, prices for that medicine were more regular and ranged from I$ 5.38 to I$ 5.49. Nonetheless, it would be somewhat naive to expect price and offer transparency from pharmaceutical companies, which may weaken their negotiating position and chance to earn more money [8, 11].

Besides, Vogler et al. [11] revealed that medicine price differences might be due to the discrepancies in economic situation between countries. They observed high prices for Austria, Germany, and Sweden and lower for Greece, Slovakia, and Hungary. It is somewhat in line with the economic situation in these countries. Nevertheless, the relationship between price level and general wealth was different for Belgium, UK, and France in their study. In our study, for instance, Austria was a middle-priced country with almost the same price for Xarelto ® and Mucosolvan ® as in Spain but with the second-lowest price for Voltaren ® among all studied countries.

The observation of lower prices in Norway than in other countries is in line with the study conducted by Brekke et al. [10] but was not revealed by Vogler et al. [1]. It may result from methodological differences between studies and the selection of medicines. Moreover, Wouters and Kanavos [4] disclosed that various methods for comparing drug prices may lead to remarkably different results. The market regulation also explains price differences between countries. Market liberalization is assumed to lead, among others, to reducing of medicines prices [21, 32, 33]. However, market deregulation in Sweden and Norway contributed mainly to the increasing number of pharmacies and longer opening hours, but price pressure was not considered as the effect of liberalization [7, 21]. It is also in line with our study, where regulated markets like Poland, Spain, or Austria were similar in prices or in some cases, cheaper than deregulated countries. Moreover, neither in Rx nor in OTC did the price comparison reveal statistical significance between regulated and deregulated markets in our study.

As we presented, medicine „A” in e.g., Romania (not listed among the wealthiest countries in the world) could be the most expensive in the analysis, but the rest of medicines were in the middle or among the cheapest offers. Such pricing irregularity also concerned the other analyzed countries like Finland or Poland. It emphasizes a need to ensure fair prices and affordability of medicines worldwide [8, 34]. Otherwise, patients may forego procurement of medicines or other essential goods or go into debt [35].

Presented drug price differences may lead to the development of a reverse traffic phenomenon, which, along with pharmaceutical supply chain disruption, deepens the risk of drug shortages in the EU, where parallel trade of goods is possible [5, 36, 37]. As we experienced during the COVID-19 pandemic, the shortage of some medicines, disinfectants, protective gloves, and masks especially during the first lockdowns, contributed to the price increase of these goods making them, in many cases, not only non-accessible but non-affordable too. Many of these goods are produced out of Europe. The COVID-19 crisis could be an opportunity to use the momentum for change. In the study by Vogler and Fisher, Switzerland and Italy mentioned that national production is needed to overcome drug shortages [6]. Moreover, bringing pharmaceutical production back to Europe has been discussed in the political debate initiated in the second half 2020 [6]. On the one hand, it may lead to a price increase, but on the other, it may contribute to better accessibility of medicines. It should also allow effective price negotiations between market stakeholders ensuring medicines at prices adequate to local economies. It is a real challenge because some prices were surprisingly high or low in our and other studies and varied significantly between or even within a country [2, 38–40].

Many countries, from the poorest to the richest, face severe cost pressures on healthcare budgets, including rising drug spending [4, 34, 40]. This, together with the global problem of high inflation rates makes the need for affordable medicines urgent for governments. It is a challenging task because many factors influence on drug prices. Besides, pharmaceutical price regulations can contribute to lowering prices and increasing healthcare access, but they reduce R&D spending [13]. All stakeholders engaged in medicine pricing and health policy should also take this into account.

### 4.1. Limitations

We are aware that the list of included countries could be expanded. Moreover, the way of getting information about the prices of selected medicines might need to be revised. For instance, presenting prices in ex-factory values without discounts could be a better way to achieve comparability in results. Nonetheless, we focused on the patients’ affordability of medicines. Thus, the decision to collect information about retail prices in local pharmacies. Apart from that, it would be valuable to investigate medicines’ price components and consider factors influencing the prices of medicines individually. These are, e.g., differences in the level of value-added tax, accessibility of generics within the market, the exact pricing, and the impact of local government institutions. It would also be interesting to present a more extensive discussion about drug prices and their affordability.

Nonetheless, discussing our results directly with other studies is difficult because we have not found drug price analyses conducted in I$. Another limitation concerns the division into regulated and deregulated markets. It might be conducted following market geography and demography. However, it may result from the pharmaceutical pricing and reimbursement systems. Some authors also indicate that markets are characterized by the mix of features of the above models, calling these markets mixed [18]. It may contribute to difficulties in uniform division into regulated and deregulated markets. Hence, there is a possibility of differences in classifying some countries as those with regulated or deregulated pharmaceutical markets.

Furthermore, according to other authors, studies on actual medicine prices need to be more comprehensive [14]. Hence, our analysis may enhance the other authors’ ability to recalculate their results to I$. Moreover, we conducted the study during the COVID-19 pandemic and related lockdowns or societal limitations. Hence, it impeded the study conduction and extended the study duration. We know that it may influence the exact assessment of the obtained results, but all prices were converted to the I$ using the rates published by the International Monetary Fund at a given time. It reduced the impact of inevitable differences in the price collected, e.g., in 2020 and 2021. Although the current study reveals the trend of the obtained results, further studies are needed to develop the results and complete conclusions despite the importance of the study.

## 5. Conclusions

Prices of medicines presented in I$ revealed differences between countries and pricing irregularities within a country. We verified that pharmaceutical market regulation does not mean higher costs of medicines. There is a need for affordable medicines, and decision-makers should focus on the prices of the medicines to make them more predictable within a country and adjust to the local economies. The study confirms that there is space for such actions. I$ could be important in pharmaceutical price analyses, and the study should encourage other researchers to present their results using this currency.

## Supporting information

S1 Checklist Human participants research checklist. (DOCX)

S1 Table Basics of pricing and reimbursement policy review in selected contries. (DOCX)

S1 Questionnaire An example of the questionnaire allowing data collection. (DOCX)

S2 Questionnaire Questionnaire on inclusivity. (DOCX)

S1 Data Base calculation sheet. (XLSX)
