# Supplementary material for: Medicines prices in International (Geary–Khamis) Dollar. The comparison between regulated and deregulated markets
Source: PLoS One. 2024 Jun 7;19(6):e0304400. doi: 10.1371/journal.pone.0304400 (PMC11161061; doi:10.1371/journal.pone.0304400)
Supplement: S1 Table — (DOCX) [file pone.0304400.s002.docx]

S1 Table. Basics of pricing and reimbursement policy review in selected contries.

|  | **Czech Republic** | **Greece** | **Romania** | **Norway** | **Switzerland** | **Japan** | **Brazil** | **USA** |
| --- | --- | --- | --- | --- | --- | --- | --- | --- |
| **Positive reimbursement list** | Yes | Yes | Yes | Yes | Yes | Yes | Yes | NA |
| **Negative reimbursement list** | No | Yes | Yes | No | No | Yes | Yes | NA |
| **Pricing** | -Follows EPR; -Reimbursement is based on the lowest price within the EU; - Products can be clustered into groups (mutually interchangeable), and reimbursement within each group is set at the lowest price of any substance within that group. | -Follows EPR; -The price of on-patent medicines is based on the average of the 3 lowest ex-factory prices among EU countries;  -The pricing of generics is based on a reference to the lowest price among 5 EU contries. | -Follows EPR; -there is an annual correction proces that reassesses aproved prices (does not apply to medicinal products authorized for special needs). | - Follows EPR;  - The Norwegian Medicines Agency is responsible for setting maximum prices on POMs; - Norway employs stepped- price system (the cost of a medicine may vary based on its therapeutic value or other factors). | - Market authorization (product cannot be introduced to the market without authorization from the competent authority) - Pharmaceutics are reimbursed if they are listed on the specialty list - Determining price (price is based on foreign price comparisons, therapeutic cross-comparisons, and marketing authorization documentation); - There are 3 conditions for reimbursement (effectiveness, appropriateness, cost-effectiveness). | - The Drug Price Standard covers all medicines necessary for medication and dispensing under the National Health Insurance;  - Pharmaceutical companies/manufacturers cannot freely set prices for medicines and medical devices covered by public health insurance;  - The Ministry of Health Labour andWelfare determines prices of medicines and medical devices;  - There are 2 methods of price setting:  a) similar efficacy comparison method b) prices of new drugs are determined based on the exitsting similar medicines or medical devices. | - The price authorization by Drug Market Regulation Chamber is obligatory for all medicines entering Brazil;  - Brazil incorporates a combination of HTA, external/internal reference pricing, and specific categorization to regulate pharmaceutical prices and ensure accessibility to essential medicines. | - Multi-payer system involves both private and public health-insurance coverage; - The pricing of drugs in the U.S. is highly dependent on the complexities of the drug supply chain; - Pharmaceutical companies set a list price for each drug. Distributors pay this price to bring the drugs to patients; -Manufacturers must ensure that drug products are available to patients, reimbursable by private or public plans, and appropriately valued for favorable coverage. |

NA – not applicable
EPR – external price referencing
VAT- value added tax
POMs – prescription only medicines
HTA – Health Technology Assessment

References to the Table 3. We accessed all positions in March and April 2024.

1. Drug Policy in the Czech Republic. <https://www.valuehealthregionalissues.com/article/S2212-1099%2817%2930059-6/pdf>.
2. Frontiers | Pharmaceutical Regulation in Central and Eastern European .... https://www.frontiersin.org/journals/pharmacology/articles/10.3389/fphar.2017.00892/full.
3. Update of Drug Pricing System in Japan - PMDA. <https://www.pmda.go.jp/files/000221888.pdf>.
4. Current Medical Technology Reimbursement System in Japan. <https://www.valuehealthregionalissues.com/article/S2212-1099%2822%2900206-0/pdf>.
5. Introduction: Understanding Japan’s Drug Pricing System. https://eradigm.com/white-paper/breaking-down-japans-drug-pricing-system/.
6. Pharmaceutical Pricing Policy in Greece: Toward a Different Path. https://www.frontiersin.org/journals/public-health/articles/10.3389/fpubh.2016.00185/full.
7. GREECE : Pharmaceutical pricing and reimbursement policies in the in .... https://ppri.goeg.at/sites/ppri.goeg.at/files/inline-files/Greece.pdf.
8. Drug Policy in Greece. https://www.valuehealthregionalissues.com/article/S2212-1099%2818%2930116-X/pdf.
9. Pharmaceutical Pricing and Reimbursement Reforms in Greece - JSTOR. <https://www.jstor.org/stable/pdfplus/40283700.pdf>.
10. OVERVIEW OF PRICING AND REIMBURSEMENT OF DRUGS REGISTRATION IN BRAZIL. https://globalregulatorypartners.com/wp-content/uploads/Pricing-and-Reimbursement-in-Brazil_04.30.20-V2.pdf.
11. Medicines regulation, pricing and reimbursement in Brazil. <https://rbfhss.org.br/sbrafh/article/view/769>.
12. Brazil Pharmaceutical pricing and reimbursement policies - goeg.at. https://ppri.goeg.at/sites/ppri.goeg.at/files/inline-files/Brazil.pdf.
13. International Reference Pricing expands reach in leading Latin American .... https://www.pharmaceutical-technology.com/pricing-and-market-access/international-referemce-pricing-expands-reach-in-leading-latin-american-countries-html/.
14. Public policy coverage and access to medicines in Brazil. https://www.scielosp.org/pdf/rsp/2022.v56/58/en.
15. Positive and negative listing | Access2Markets - europa.eu. https://trade.ec.europa.eu/access-to-markets/en/content/positive-and-negative-listing.
16. Pricing and reimbursement of pharmaceuticals in Norway. https://link.springer.com/article/10.1007/s10198-002-0135-4.
17. The Key to Understanding Pricing and Reimbursement in the Nordics. https://www.propharmagroup.com/thought-leadership/understanding-pricing-and-reimbursement-nordics.
18. Public funding and pricing - Legemiddelverket. https://legemiddelverket.no/english/public-funding-and-pricing.
19. Positive and negative listing | Access2Markets - europa.eu. https://trade.ec.europa.eu/access-to-markets/en/content/positive-and-negative-listing.
20. Overview of reimbursable healthcare in other EU/EEA countries. https://www.helsenorge.no/en/treatment-abroad/overview-of-reimbursable-healthcare/.
21. Report from: Romania - pharmtech.com. https://www.pharmtech.com/view/report-romania.
22. Market Access & Health Technology Assesment: Romania. https://pharmaboardroom.com/legal-articles/regulatory-pricing-and-reimbursement-romania/.
23. Pricing & Reimbursement 2018. <https://bsmp.ro/wp-content/uploads/2019/01/GLI-PR1_Romania.pdf>.
24. Themed Section: Drug Policies in Central and Eastern Europe. https://www.valuehealthregionalissues.com/article/S2212-1099%2818%2930052-9/pdf.
25. National Agency for Medicines and Medical Devices of Romania. <https://www.anm.ro/en/>.
26. Pricing of Medicines in Switzerland - CMS LAW-NOW. https://cms-lawnow.com/en/ealerts/2023/01/pricing-of-medicines-in-switzerland-three-yearly-price-review-what-are-the-most-important-changes-for-2023.
27. PHARMACEUTICAL PRICING AND REIMBURSEMENT POLICIES IN SWITZERLAND - OECD. https://www.oecd.org/switzerland/38868953.pdf.
28. Pricing & Reimbursement Laws and Regulations | Switzerland | GLI. <https://www.globallegalinsights.com/practice-areas/pricing-and-reimbursement-laws-and-regulations/switzerland>.
29. Pricing & Reimbursement Laws and Regulations | USA | GLI. https://www.globallegalinsights.com/practice-areas/pricing-and-reimbursement-laws-and-regulations/usa.
30. The Need to Treat the Ailing U.S. Pharmaceutical Pricing System. https://www.hsph.harvard.edu/ecpe/united-states-pharmaceutical-pricing/.
31. Pricing of pharmaceuticals is becoming a major challenge for health systems. <https://www.bmj.com/content/368/bmj.l4627>.
32. General Overview of US HEALTHCARE Reimbursement Systems - IDR Medical. https://info.idrmedical.com/blog/usa-medical-device-reimbursement.
33. The US Healthcare Reimbursement System Summary. https://healthcaredata.center/government-regulation/the-us-healthcare-reimbursement-system-summary/.
