# Supplementary material for: Medicines prices in International (Geary–Khamis) Dollar. The comparison between regulated and deregulated markets
Source: PLoS One. 2024 Jun 7;19(6):e0304400. doi: 10.1371/journal.pone.0304400 (PMC11161061; doi:10.1371/journal.pone.0304400)
Supplement: S1 Questionnaire — (DOCX) [file pone.0304400.s003.docx]

|  | Price 1 | Price 2 | Price 3 |
| --- | --- | --- | --- |
| Mysimba ® |  |  |  |
| Cardura XL 4 mg 30 pills ® |  |  |  |
| Yasmin ® |  |  |  |
| Preductal MR 35 mg 60 pills ® |  |  |  |
| Xarelto 20 mg 28 pills® |  |  |  |
| Aspirin C 10 soll. Tabs ® |  |  |  |
| Voltaren gel 50g ® |  |  |  |
| Nurofen express 400 mg 20 pills ® |  |  |  |
| Tantum verde spray 1,5mg/ml 30 ml® |  |  |  |
| Mucoslovan 6mg/ml 100 ml ® |  |  |  |

Country:
